# The efficiency of liposomal paclitaxel versus docetaxel in neoadjuvant chemotherapy with the TPF regimen for locally advanced nasopharyngeal carcinoma: a retrospective study

**DOI:** 10.3389/fonc.2024.1465038

**Published:** 2024-10-10

**Authors:** Zhi Yang, Quan Zuo, Rong Liu, Hui Wu, Jia Chen, Li Xiong, Jieqi Jia, Zhibi Xiang

**Affiliations:** ^1^ Department of Oncology, People’s Hospital of Xiangxi Tujia and Miao Autonomous Prefecture, First Affiliated Hospital of Jishou University, Jishou, Hunan, China; ^2^ Department of Otolaryngology, People’s Hospital of Xiangxi Tujia and Miao Autonomous Prefecture, First Affiliated Hospital of Jishou University, Jishou, Hunan, China

**Keywords:** nasopharyngeal carcinoma, neoadjuvant chemotherapy, paclitaxel liposomes, docetaxel, TPF regimen

## Abstract

**Purpose:**

This retrospective study aimed to explore the efficiency and untoward reaction of liposomal paclitaxel versus docetaxel for locally advanced nasopharyngeal carcinoma (NPC).

**Methods:**

This retrospective study included 115 patients diagnosed with NPC at our hospital between January 2018 and December 2021. Patients were stratified into two groups based on their treatment with either liposomal paclitaxel (*n* = 71) or docetaxel (*n* = 44) as part of the neoadjuvant chemotherapy regimen. Objective response rate (ORR), progression-free survival (PFS), locoregional relapse-free survival (LRFS), distant metastasis-free survival (DMFS), and overall survival (OS) were compared between the two groups.

**Results:**

ORR was significantly improved in the liposomal paclitaxel group than in the docetaxel group (62.0% versus 40.9%, *p* = 0.028). The 3-year PFS (PFS: 84.4% versus 77.5%, *p* = 0.303), LRFS (95.8% versus 94.4%, *p* = 0.810), DMFS (87.2% versus 83.0%, *p* = 0.443), and OS (90.7% versus 88.8%, *p* = 0.306) revealed no significance. The neutrophil-to-lymphocyte ratio [hazard ratio (HR): 3.510; *p* = 0.039] and distant metastasis (HR: 4.384; *p* = 0.035) were regarded as the risk factors using multivariate regression analysis. Moreover, the incidence of leukopenia at grades 1–2 in the liposomal paclitaxel group was significantly lower than that in the docetaxel group (28.1% versus 79.5%, *p* < 0.05).

**Conclusions:**

Liposomal paclitaxel had better efficacy in terms of short-term effects and lower incidence of leukopenia at grades 1–2 compared with the docetaxel group.

## Introduction

Nasopharyngeal carcinoma (NPC) is one of the common malignant tumors in the head and neck, with a unique and uneven geographical distribution. It is extremely prevalent in southern China, particularly in Guangdong, Guangxi, Fujian, Hunan, and other southern provinces (1, 2). Approximately 70%–85% of patients with NPC are already in the locally advanced stage at the first diagnosis (3). Synchronous radiotherapy and chemotherapy is the main treatment for patients with locally advanced NPC at present (4). Previous studies (5–7) have reported improved efficacy of sequential concurrent radiotherapy and chemotherapy after neoadjuvant chemotherapy for locally advanced NPC. Docetaxel, cisplatin, and 5-fluorouracil (TPF) are commonly utilized as first-line neoadjuvant chemotherapy regimens for locally advanced NPC, but still with many adverse reactions (8). Additionally, as a novel type of paclitaxel preparation, liposomal paclitaxel could reduce its toxicity and improve bioavailability (9), which has similar antitumor efficacy compared to traditional paclitaxel (10). Thus, the use of liposome-based paclitaxel in TPF neoadjuvant chemotherapy for locally advanced NPC may enhance treatment efficacy and mitigate adverse reactions? This study retrospectively analyzed the efficacy and adverse reactions of paclitaxel liposomes versus docetaxel in neoadjuvant chemotherapy for locally advanced NPC.

## Materials and methods

### General information

This study included 115 patients with NPC admitted to our hospital from January 2018 to December 2021. The inclusion criteria were as follows: (1) patients with NPC aged 18–70 years; (2) patients pathologically confirmed as NPC [including non-keratinizing carcinoma (differentiated and undifferentiated), keratinizing squamous cell carcinoma, basal cell squamous cell carcinoma, and adenocarcinoma, and excluding neuroendocrine carcinoma]; (3) NPC diagnosed as T1-2N2M0, T3N1-2M0, T1-3N3M0, and T4N0-3M0 based on imaging examination (the eighth version); and (4) patients receiving neoadjuvant chemotherapy with liposomal paclitaxel or TPF, followed by sequential radical synchronous radiotherapy and chemotherapy.

### Treatment

Patients were divided into the liposomal paclitaxel and docetaxel groups. All patients were treated with TPF regimen neoadjuvant chemotherapy after two cycles of sequential radical radiotherapy and chemotherapy. Patients with NPC in the liposomal paclitaxel group were treated as follows: paclitaxel liposome at 135 mg/m^2^ on day 1, cisplatin at 25 mg/m^2^ on days 1–3, and fluorouracil at 600/m^2^ on continuous intravenous infusion on days 1–5, repeated every 3 weeks. Patients in the docetaxel group were treated with docetaxel at 60 mg/m^2^ on day 1, with other treatments consistent with the liposomal paclitaxel group. Patients in both groups underwent synchronous radiotherapy and chemotherapy 1–2 weeks after the completion of neoadjuvant chemotherapy using three-dimensional conformal intensity-modulated radiotherapy. The prescribed dose parameters were as follows: NPC gross tumor volume (GTV) of 69.96–73.92 Gy/33f; cervical node GTV of 69.96 Gy/33f; clinical target volume (CTV1) of 60.06 Gy/33f; and CTV2 of 50.4 Gy/28f. Patients receive radiotherapy once a day, from Monday to Friday, and rest on Saturdays and Sundays. A single cisplatin dose was administered simultaneously during radiotherapy. Specifically, cisplatin at 80 mg/m^2^ was administered for 21 days in 3 days for two cycles.

### Efficacy and observation indicators

Short-term efficacy: The maximum diameter of the tumor was measured using imaging examination, which was evaluated through response evaluation criteria in solid tumor (version 1.1). The efficacy determinations included complete response (CR), partial response (PR), stable disease (SD), and progressive disease (PD). Additionally, objective response rate (ORR) was an efficacy determination, which consists of CR and PR. The short-term efficacy was assessed 1 week after the completion of the second cycle of neoadjuvant chemotherapy.

Short-term toxicity response: Toxicity was recorded during treatment that was evaluated using common terminology criteria for adverse events (version 3).

Long-term efficacy: Local recurrence, distant metastasis, and survival status were confirmed by following all patients. The primary endpoints included progression-free survival (PFS), local relapse-free survival (LRFS), and distant metastasis-free survival (DMFS). The secondary endpoint was overall survival (OS). The long-term efficacy assessment began on the first day of the first cycle of neoadjuvant chemotherapy and continued until the end of the follow-up period or the time of occurrence of a positive event.

### Follow-up

Follow-up was performed every 3 months within 2 years after the end of radical chemoradiotherapy, every 6 months within 2 to 5 years after the end, and every 1 year after 5 years. All patients were followed up until 28 February 2023. The follow-up period was 11–64 months, with a median of 36.0 months.

### Statistical analysis

All data were analyzed using version 22.0 of SPSS. Categorical data were expressed as rates and were tested using the chi-square test, the continuity correction chi-square test, or Fisher’s exact test as appropriate. We estimated survival curves and rates for time-to-event endpoints (overall survival, progression-free survival, relapse-free survival, and distant metastasis-free survival) using the Kaplan–Meier method. We compared survival between the two treatment groups using stratified log-rank tests. We performed Cox proportional hazards regression analysis for various factors including age (<60 years vs. ≥60 years), gender (male vs. female), pathological type (non-keratinizing carcinoma vs. keratinizing carcinoma), clinical stage (stage III vs. stage IV), T stage (T1-2 vs. T3-4), N stage (N0-1 vs. N2-3), Epstein–Barr Virus (EBV) DNA level (<100 copies/mL vs. ≥100 copies/mL), granulocyte/lymphocyte ratio (>4 vs. ≤4), lactate dehydrogenase (LDH, >245 U/L vs. ≤245 U/L), hemoglobin (<120 g/L vs. ≥120 g/L), epidermal growth factor receptor (EGFR) expression (positive vs. negative), and treatment method (liposomal paclitaxel vs. docetaxel). The incidence of adverse reactions was tested using the chi-square test, the continuity correction chi-square test, or Fisher’s exact test as appropriate. *p*-values of <0.05 were considered significant.

## Results

**Figure 1:**
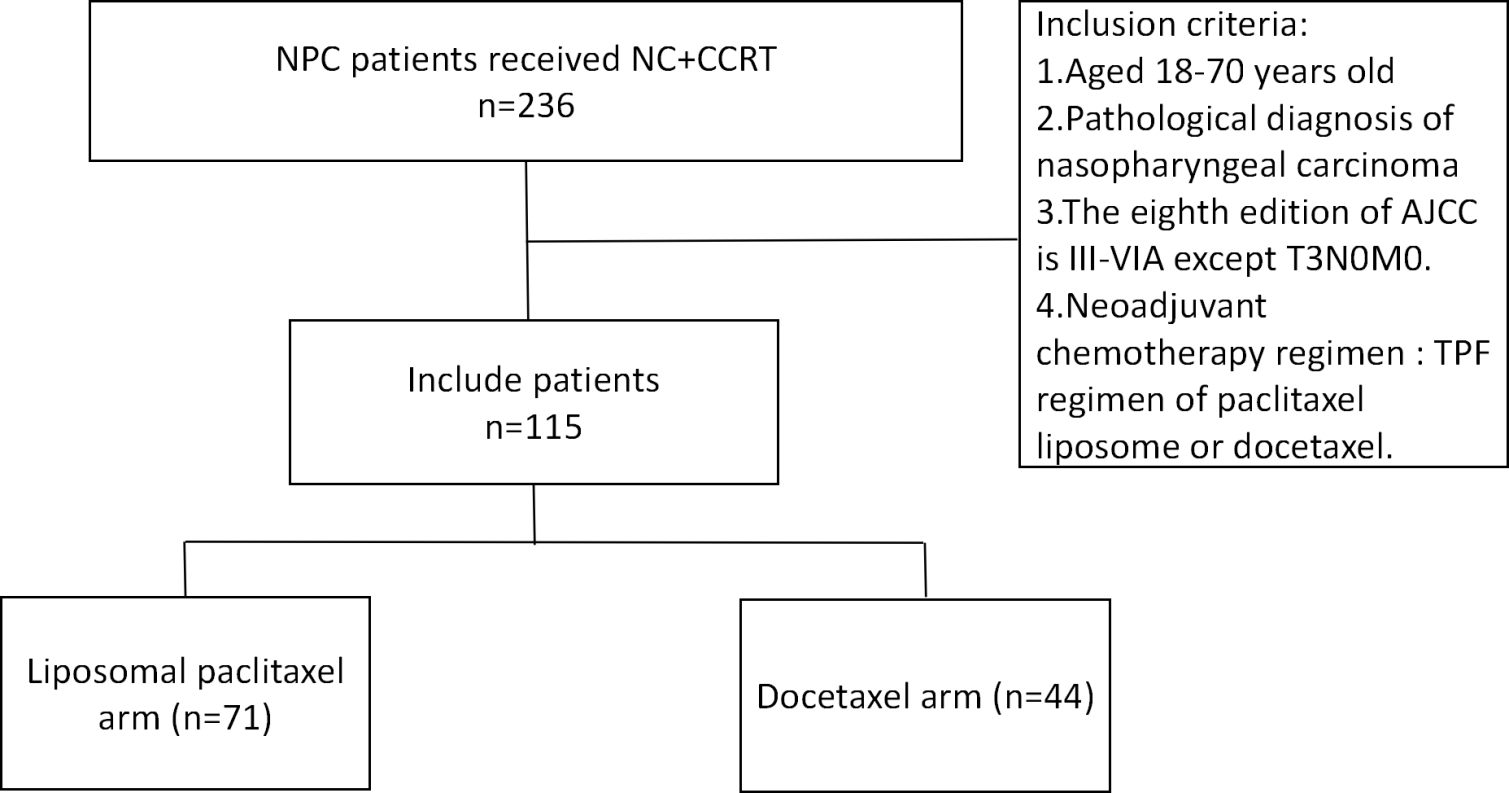
Flowchart for patient inclusion. NPC, nasopharyngeal carcinoma; NC, neoadjuvant chemotherapy; CCRT, concurrent chemoradiotherapy; AJCC, American Joint Committee on Cancer; TPF, docetaxel, cisplatin, and 5-fluorouracil.

From January 2018 to December 2021, 115 patients were screened out to meet the conditions of this study. In the NC regimen, there were 71 cases (61.7%) in the liposomal paclitaxel group and 44 cases (38.3%) in the docetaxel group. Patients in both groups completed 2 cycles of neoadjuvant chemotherapy and concurrent chemoradiotherapy (**Figure 1**).

### Comparison of baseline data between liposomal paclitaxel and docetaxel groups

Significant differences in age, gender, pathological type, clinical stage, T stage, N stage, EBV DNA level, neutrophil-to-lymphocyte ratio (NLR), LDH, hemoglobin, and epithelial growth factor receptor (EGFR) expression were not observed between the two groups, as shown in **Table 1** (*p* > 0.05).

**Table 1:** Comparison of baseline characteristics between the two groups.

Characteristic	Paclitaxel liposome	Docetaxel	χ^2^ value	*p*-value
Age (years)			1.833	0.176
<60	58 (71.7)	40 (81.9)		
≥60	13 (18.3)	4 (9.1)		
M ± SD	49.59 ± 9.69	49.32 ± 7.79		
Gender			0.001	0.981
Male	55 (77.5)	34 (77.3)		
Female	16 (22.5)	10 (22.7)		
Pathological type			0.211	0.646
Non-keratinizing carcinoma	66 (93.0)	39 (88.6)		
Keratocarcinoma	5 (7.0)	5 (11.4)		
Basal cell carcinoma	0 (0.0)	0 (0.0)		
Clinical stage			0.041	0.839
III	14 (19.7)	8 (18.2)		
IVa	57 (80.3)	36 (81.8)		
T stage			1.725	0.631
T1	4 (5.6)	1 (2.3)		
T2	7 (9.9)	4 (9.1)		
T3	24 (33.8)	12 (27.3)		
T4	36 (50.7)	27 (61.4)		
N stage			4.237	0.120
N0	0 (0.0)	0 (0.0)		
N1	1 (1.4)	4 (9.1)		
N2	34 (47.9)	22 (50.0)		
N3	36 (50.7)	18 (40.9)		
EB-DNA (copies/mL)			0.467	0.495
<100	23 (32.4)	17 (38.6)		
≥100	48 (67.6)	27 (61.4)		
Granulocyte/Lymphocyte ratio			0.454	0.500
>4	7 (9.9)	2 (4.5)		
≤4	64 (90.1)	42 (95.5)		
LDH (U/L)			0.001	0.975
>245	3 (4.2)	1 (2.3)		
≤245	68 (95.8)	43 (97.7)		
HB (g/L)				
<120	9 (12.7)	5 (11.4)	0.440	0.834
≥120	62 (87.3)	39 (88.6)		
EGFR expression			0.608	0.436
Negative	3 (4.2)	0 (0.0)		
Positive	68 (95.8)	44 (100.0)		

EB-DBA, Epstein–Barr virus deoxyribonucleic acid; EB-DNA < 100 copies/mL is negative, ≥ 100 copies/mL is positive. M, mean; SD, standard deviation; LDH, lactate dehydrogenase; HB, hemoglobin; EGFR, epithelial growth factor receptor.

### Comparison of short-term efficacy between the liposomal paclitaxel and docetaxel groups

The paclitaxel liposome and docetaxel groups had 0, 44, 27, and 0 cases and 0, 18, 26, and 0 cases with CR, PR, SD, and PD, with an ORR of 62.0% (44/71) and 40.9% (18/44), respectively. The ORR was significantly higher in the paclitaxel liposome group than in the docetaxel group (χ² = 4.850, *p* = 0.028). Among the 115 patients, one patient in the docetaxel group had SD with tumor enlargement, while the rest had SD with tumor shrinkage. All patients with SD in the paclitaxel liposome group had tumor shrinkage (**Table 2**).

**Table 2:** Comparison of short-term efficacy between the two groups.

Groups	Total cases	CR	PR	SD	PD	ORR
		Cases (%)	Cases (%)	Cases (%)	Cases (%)	Cases (%)
Paclitaxel liposome	71	0 (0.0)	44 (62.0)	27 (38.0)	0 (0.0)	44 (62.0)
Docetaxel	44	0 (0.0)	18 (40.9)	26 (59.1)	0 (0.0)	18 (40.9)
χ^2^ value		–	4.850	4.850	–	4.850
*p-*value		–	0.028	0.028	–	0.028

CR, complete response; PR, partial response; SD, stable disease; PD, progressive disease; ORR, objective response rate. - means unable to calculate statistics.

### Comparison of long-term efficacy between the liposomal paclitaxel and docetaxel groups

**Figure 2:**
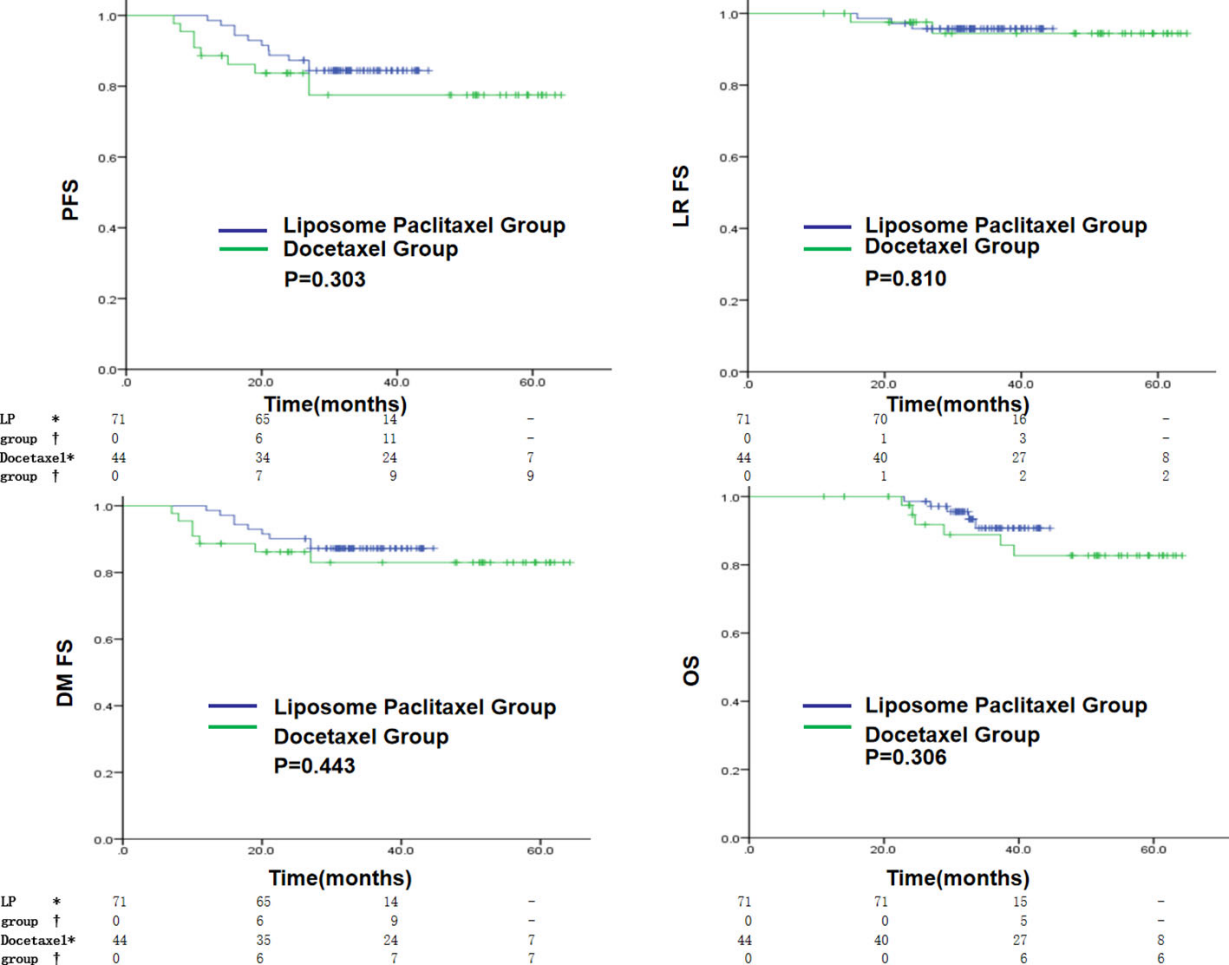
Kaplan–Meier curves in the initial cohort with a liposomal paclitaxel-based TPF regimen or a docetaxel-based TPF regimen for PFS, LRFS, DMFS and OS. * indicates the number of censored individuals, † indicates the number of positive individuals, - indicates that the patient’s follow-up time did not reach that time point, LP refers to paclitaxel liposome.

The liposome paclitaxel group had three, nine, and five cases and the docetaxel group had two, seven, and six cases of local recurrence, distant metastasis, and death, respectively. Overall, no significant difference in 3-year PFS, LRFS, DMFS, and OS was found between the two groups (PFS: 84.4% vs. 77.5%, *p* = 0.303; LRFS: 95.8% vs. 94.4%, *p* = 0.810; DMFS: 87.2% vs. 83.0%, *p* = 0.443; OS: 90.7% vs. 88.8%, *p* = 0.306) (**Figure 2**).

The multivariate logistic regression analysis revealed NLR as the independent risk factor of the disease progression [hazard ratio (HR) = 3.510, *p* = 0.039] and distant metastasis (HR = 4.384, *p* = 0.035) of locally advanced NPC (**Table 3**). The higher the NLR, the higher risks the disease progression and distant metastasis had in patients with locally advanced NPC.

**Table 3:** Multivariate regression analysis.

Clinical factors	Hazard ratio (95% CI)	*p*-value
PFS		
Gender (male vs. female)	0.807 (0.225–2.898)	0.742
Age	1.333 (0.716–2.4479)	0.364
Granulocyte/Lymphocyte ratio	3.510 (1.067–11.548)	0.039
Treatment (paclitaxel liposome vs. docetaxel)	1.914 (0.743–4.931)	0.179
OS		
Gender (male vs. female)	1.740 (0.333–9.099)	0.512
Age	1.866 (0.754–4.617)	0.177
Granulocyte/Lymphocyte ratio	4.046 (0.706–23.192)	0.117
Treatment (paclitaxel liposome vs. docetaxel)	3.002 (0.666–13.654)	0.155
LRFS		
Gender (male vs. female)	1.394 (0.055–35.081)	0.840
Age	0.897 (0.182–4.416)	0.893
Granulocyte/Lymphocyte ratio	1.962 (0.138–27.863)	0.619
Treatment (paclitaxel liposome vs. docetaxel)	2.553 (0.255–25.532)	0.425
DMFS		
Gender (male vs. female)	0.349 (0.066–1.838)	0.214
Age	2.065 (0.963–4.425)	0.062
Granulocyte/Lymphocyte ratio	4.384 (1.110–17.312)	0.035
Treatment (paclitaxel liposome vs. docetaxel)	1.547 (0.483–4.958)	0.463

CI, confidence interval. The Cox proportional hazards regression model was used to analyze variables, including age (<60 years vs. ≥60 years), gender (male vs. female), pathological type (non-keratinizing carcinoma vs. keratinizing carcinoma), clinical stage (stage III vs. stage IV), T stage (T1–2 vs. T3–4), N stage (N0–1 vs. N2–3), Epstein–Barr Virus (EBV) DNA level (<100 copies/mL vs. ≥100 copies/mL), granulocyte/lymphocyte ratio (>4 vs. ≤4), lactate dehydrogenase (LDH, >245 U/L vs. ≤245 U/L), hemoglobin (<120 g/L vs. ≥120 g/L), epidermal growth factor receptor (EGFR) expression (positive vs. negative), and treatment method (liposomal paclitaxel vs. docetaxel).

### Adverse events

The incidence of grade 1–2 leukopenia was significantly lower in the liposome paclitaxel than in the docetaxel group (*p* < 0.05, **Table 4**). Other adverse events, including grade 3–4 leukopenia, neutropenia, increased alanine transaminase, increased aspartate aminotransferase, nausea, vomiting, peripheral neuropathy, diarrhea, and constipation, had no significant differences between the two groups (*p* > 0.05).

**Table 4:** Comparison of adverse reactions between the two groups.

Adverse reactions	Paclitaxel liposome (*n* = 71)		Docetaxel (*n* = 44)		*p*1-value	*p*2-value
	1-2 (%)	3-4 (%)	1-2 (%)	3-4 (%)		
Leukopenia	20 (28.1)	2 (2.8)	35 (79.5)	2 (9.1)	0.000	1.000
Neutropenia	3 (4.2)	0 (0.0)	1 (2.3)	0 (0.0)	0.975	–
Hemoglobin decreased	5 (7.0)	0 (0.0)	3 (6.8)	0 (0.0)	1.000	–
Thrombocytopenia	3 (4.2)	0 (0.0)	2 (4.5)	0 (0.0)	1.000	–
ALT increased	42 (59.2)	0 (0.0)	25 (56.8)	0 (0.0)	0.805	–
AST increased	9 (12.7)	0 (0.0)	3 (6.8)	0 (0.0)	0.493	–
Urea increased	9 (12.7)	0 (0.0)	7 (15.9)	0 (0.0)	0.626	–
Nausea	43 (60.6)	0 (0.0)	29 (65.9)	0 (0.0)	0.565	–
Vomiting	9 (12.7)	0 (0.0)	7 (15.9)	0 (0.0)	0.626	–
Peripheral neuropathy	0 (0.0)	0 (0.0)	1 (2.3)	0 (0.0)	–	–
Diarrhea	1 (1.4)	0 (0.0)	2 (4.5)	0 (0.0)	0.672	–
Constipation	5 (7.0)	0 (0.0)	6 (13.6)	0 (0.0)	0.400	–

ALT, alanine aminotransferase; AST, aspartate aminotransferase. p1 is the p-value of the chi-square test for grade 1–2 adverse reactions, and p2 is the p-value of the chi-square test for grade 3–4 adverse reactions; - means unable to calculate statistics.

## Discussion

Distant metastasis is a leading cause of ineffective treatment of locally advanced NPC (11). Previous studies have revealed that neoadjuvant chemotherapy can eradicate micro-metastatic lesions and relieve symptoms while having good compliance (12). Studies have demonstrated improved curative effect and reduced distant metastasis with sequential concurrent chemoradiotherapy following neoadjuvant chemotherapy (5, 6, 13), thereby becoming the standard therapy for locally advanced NPC. The TPF regimen has been recommended (grade I) for neoadjuvant chemotherapy (14); however, it has certain toxic side effects such as leukopenia and gastrointestinal reactions (15).

Liposomal paclitaxel is a novel paclitaxel drug encapsulated with liposomes. As novel drug carriers, liposomes improve the solubility of paclitaxel with advantages, including ease of synthesis and good biocompatibility (16, 17), leading to reduced toxicity and improved bioavailability (9). Su et al. have revealed that liposomal paclitaxel has comparable antitumor efficacy with docetaxel and higher safety in treating breast cancer (18). However, few studies have focused on the comparison of the efficacy and toxicity of liposomal paclitaxel with that of docetaxel in NPC.

The results of this study show that compared with the docetaxel group, the liposomal paclitaxel group had a better objective response rate, indicating that liposomal paclitaxel has better short-term efficacy in the treatment of locally advanced NPC, and these better objective response rates may translate into higher overall survival benefits during long-term follow-up. Liposomal paclitaxel had comparable long-term effects with docetaxel, whereas it had a lower incidence of grade 1–2 leukopenia in terms of adverse effects. Liu et al. (19) observed similar results regarding the long-term effect in a retrospective study involving 767 cases of locally advanced NPC. However, they did not pay attention to the short-term effect. Additionally, Liu et al. revealed that liposomal paclitaxel had a higher incidence of grade 3–4 leukopenia and neutropenia, which may be attributed to the three to four cycles of neoadjuvant chemotherapy in the study, whereas our research utilized a neoadjuvant chemotherapy regimen consisting of only two cycles. Currently, the optimal course of neoadjuvant chemotherapy remains debated. Chang et al. conducted a retrospective study involving 959 cases of two to three locally advanced NPC and revealed that ≥3 cycles of neoadjuvant chemotherapy followed by radiotherapy had a comparable curative effect with that of concurrent radiochemotherapy. However, the former had fewer adverse effects, including myelosuppression, nausea, vomiting, oral mucositis, and xerostomia (15). These findings indicated that ≥3 cycles of neoadjuvant chemotherapy may not improve the curative effect but increase the adverse effects in treating locally advanced NPC.

The cumulative dose of cisplatin in concurrent radiochemotherapy is also debated. Lv et al. revealed no significant association between a 200 mg/m^2^ cumulative dose of cisplatin and improved survival, while a 160 mg/m^2^ cumulative dose of cisplatin may be appropriate (20). Liu et al. revealed a better curative effect with a >200 mg/m^2^ than ≤100 mg/m^2^ cumulative dose of cisplatin, but was comparable with 100–200 mg/m^2^ cumulative dose in concurrent radiochemotherapy (21). These findings suggested that the higher cumulative dose of cisplatin did not always indicate a better curative effect. The present study adopted a 160 mg/m^2^ cumulative dose of cisplatin. In contrast, some studies have revealed a better survival benefit with a higher cumulative dose of cisplatin. Jiang et al. revealed higher 3-year PFS and DMFS in the >200 mg/m^2^ cumulative dose of cisplatin than that in the ≤200 mg/m^2^ cumulative dose in concurrent radiochemotherapy (22).

NLR was identified as the independent risk factor of the disease progression and distant metastasis of locally advanced NPC in multivariate logistic regression analysis. NLR is the ratio of the neutrophil and lymphocyte absolute counts in peripheral blood, representing the balance between pro-tumor inflammatory and antitumor immune response. Several studies have proved NLR as an independent prognostic factor of tumors, confirming our results (23–26). Other prognostic factors (27–32) were negative in the present study, including EGFR, LDH, T stage, and N stage, which may be associated with the small sample size and short follow-up visit.

NPC therapy aimed to improve OS and reduce toxic and side effects. Therefore, drug efficacy and patient tolerance should be considered regarding the choice of neoadjuvant chemotherapy. Our study provides the clinical evidence to support the use of the liposomal paclitaxel-based TPF regimen. However, this study has several limitations. This study is a single-center, retrospective study with a relatively short follow-up period for patients in the liposomal paclitaxel group, which has limited the acquisition of more survival data. Additionally, adverse reactions such as allergic reactions and alopecia caused by paclitaxel were not collected for either group. These factors may introduce certain biases to our research findings. Multi-center, prospective, randomized controlled studies are required in the future to confirm our results.

## Conclusion

In conclusion, liposome paclitaxel exhibited similar long-term effects compared with docetaxel in neoadjuvant chemotherapy for locally advanced NPC, while it possessed better short-term effects and a lower prevalence of grade 1–2 leukopenia. The liposomal paclitaxel-based TPF regimen may serve as a promising alternative therapeutic strategy to the docetaxel-based TPF regimen in patients with locally advanced NPC.

## Data Availability

The original contributions presented in the study are included in the article/supplementary material. Further inquiries can be directed to the corresponding authors.
